# Characterization of the Plastic Scintillator Detector System Exradin W2 in a High Dose Rate Flattening-Filter-Free Photon Beam

**DOI:** 10.3390/s22186785

**Published:** 2022-09-08

**Authors:** Sara Thrower, Surendra Prajapati, Shannon Holmes, Emil Schüler, Sam Beddar

**Affiliations:** 1 Department of Radiation Physics, The University of Texas MD Anderson Cancer Center, Houston, TX 77030, USA; 2 Medical Physics Program, The University of Texas MD Anderson UTHealth Graduate School of Biomedical Sciences, Houston, TX 77030, USA; 3 Standard Imaging Inc., Middleton, WI 53562, USA

**Keywords:** Exradin W2, plastic scintillator, flattening-filter-free, high dose rate, output constancy, high dose per pulse, dosimetry

## Abstract

(1) Background: The Exradin W2 is a commercially available scintillator detector designed for reference and relative dosimetry in small fields. In this work, we investigated the performance of the W2 scintillator in a 10 MV flattening-filter-free photon beam and compared it to the performance of ion chambers designed for small field measurements. (2) Methods: We measured beam profiles and percent depth dose curves with each detector and investigated the linearity of each system based on dose per pulse (DPP) and pulse repetition frequency. (3) Results: We found excellent agreement between the W2 scintillator and the ion chambers for beam profiles and percent depth dose curves. Our results also showed that the two-voltage method of calculating the ion recombination correction factor was sufficient to correct for the ion recombination effect of ion chambers, even at the highest DPP. (4) Conclusions: These findings show that the W2 scintillator shows excellent agreement with ion chambers in high DPP conditions.

## 1. Introduction

Flattening-filter-free (FFF) photon beams are becoming increasingly common for the delivery of intensity-modulated radiation therapy in the clinic [1]. Flattening-filter-free beams are delivered without the use of a flattening filter, which results in a peaked beam profile and dose rates approximately four times higher than those of flattened beams. Prior work has shown that the cylindrical ion chambers typically used for scanning and reference dosimetry begin to experience non-negligible recombination effects at these higher dose rates, which must be accounted and corrected for [2]. Lang et al. [3] investigated five commonly used ionization chambers in a 10 MV FFF photon beam and found that the collection efficiencies range from 0.994 for an Advanced Markus chamber to 0.988 for a Semiflex ion chamber. Kry et al. [2] measured a recombination correction factor (P_ion_) of three farmer-type chambers as high as 1.018 for 10 MV FFF beams at 100 cm source-to-surface distance (SSD) at the depth of maximum dose (d_max_). Accurate determination of ion recombination correction factors adds extra time and effort to the already laborious task of beam characterization.

Plastic scintillator detectors provide a potential solution to this problem because of their high yield of light emission [4,5,6,7]. When irradiated, plastic scintillators emit scintillation light proportional to the energy absorbed [8]. An optical light guide transmits the light to a photodetector that converts the light into an electrical signal for analysis [9]. Plastic scintillators are water equivalent, waterproof, energy-independent within the megavoltage range, unaffected by pressure, and less sensitive to radiation damage than diodes [10,11]. They also have a high spatial resolution, provide very reproducible and very stable measurements [8,12], and have been shown to be linear with dose rate [13,14,15,16]. However, one drawback of scintillator detector systems is the stem effect arising from the collection of Cerenkov light produced in the light guide, which results in an artificially high reading that must be accounted for [17,18].

The Exradin W2 (Standard Imaging, Inc., Middleton, WI, USA) is a commercially available plastic scintillator detector system; its predecessor was the W1 scintillator, which was developed specifically for depth- and lateral-beam-profile scanning, as well as relative dosimetry, such as output determination [16]. The W2 scintillator detector system uses the chromatic spectral technique described by Guillot et al. [19] to correct for the Cerenkov light contribution. The W2 scintillator has previously been characterized in small fields of flattened and unflattened beams [4,5,6,7,20]. In this work, we conducted scanning and dosimetric measurements with the W2 scintillator detector system in an FFF beam and compared them to the CC01 and CC08 ion chambers (IBA Dosimetry GmBH, Schwarzenbruck, Germany) designed for small-field dosimetry. We chose to compare the W2 to the CC08 ion chamber for PDD measurements because the CC08 is large enough to have minimal polarity effects at the surface and is small enough to be largely unaffected by volume averaging [21].

## 2. Materials and Methods

**Figure 1:**
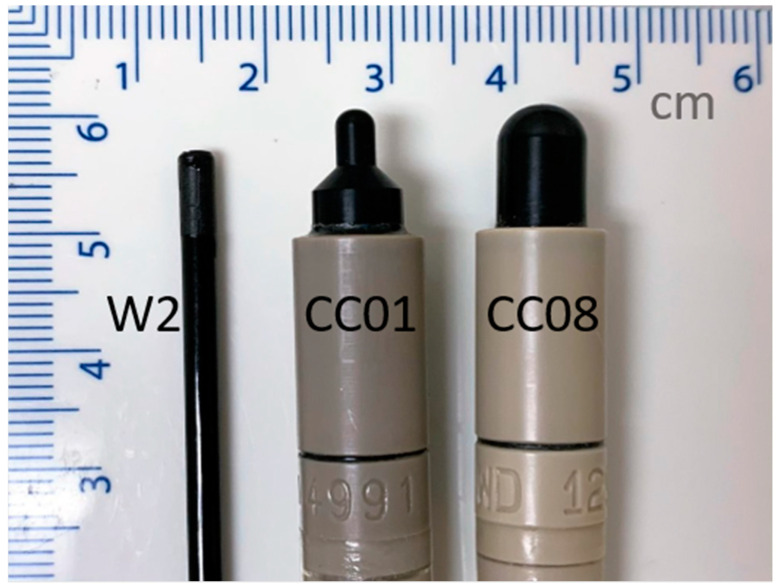
Exadrin W2 Scintillator from Standard Imaging (**left**) and the IBA Dosimetry CC01 (**center**) and CC08 (**right**) ion chambers.

Measurements were performed on a Varian TrueBeam linear accelerator (Varian Medical Systems, Palo Alto, CA, USA) using a 10 MV FFF beam, commissioned to deliver 1 cGy/MU to water at dmax for a 10 cm × 10 cm field at a 100 cm SSD [22]. Percentage depth dose (PDD) curves, lateral profiles, and output factors as a function of field size and dose per pulse (DPP) were measured with the W2 scintillator. For comparison, the PDDs and profiles were obtained with a CC01 ionization chamber, and the output factors with both a CC01 and CC08 ionization chamber. Profile measurements were acquired using a 3D Scanner scanning tank, and PDDs and output factors were acquired in a 1D Scanner tank (Sun Nuclear Corporation, Melbourne, FL, USA). The detectors are shown in Figure 1, and the physical properties of each detector are presented in Table 1.

We characterized the recombination (P_ion_) and polarity (P_pol_) correction factors of the CC08 and CC01 ion chambers in a water phantom at a depth of maximum dose at 100 cm, 90 cm, and 80 cm SSD using the two-voltage method [22]. We delivered 200 MU at 2400 MU/min nominal dose rate for the recombination and polarity correction measurements. For these measurements, an ADCL calibrated CNMC-206 electrometer (CNMC Co. Inc, Nashville, TN, USA) and a DOSE 1 electrometer (IBA Dosimetry) were both used at −300 V bias.

The W2 scintillator detector was characterized according to the manufacturer-suggested Cerenkov-to-light ratio (CLR) correction procedure in a water tank using the rectangular field method. The procedure is described in detail by Galavis et al. [20]. In short, two measurements were taken, one with a large length of stem in the field and one with a minimal length of stem in the field [19]. The collected light was spectrally separated into a blue channel and a green channel. The CLR was calculated according to:

(1) $$\mathrm{CLR}=\frac{{\mathrm{Blue}}_{\mathrm{Max}}-{\mathrm{Blue}}_{\mathrm{Min}}}{{\mathrm{Green}}_{\mathrm{Max}}-{\mathrm{Green}}_{\mathrm{Min}}},$$

where Blue_Min_, Blue_Max_, Green_Min_, and Green_Max_ are the magnitudes of light from the blue and green channels in the minimum and maximal stem-irradiated configurations, respectively. The CLR was accounted for in the measurements according to:

(2) $$\mathrm{Signal}={\mathrm{Blue}}_{\mathrm{Meas}}-\left({\mathrm{Green}}_{\mathrm{Meas}}\;*\;\mathrm{CLR}\right),$$

where Blue_Meas_ and Green_Meas_ are the magnitude of light measured from the blue and green channel, respectively.

PDD curves were collected with the CC01 ion chamber and W2 scintillator at 100 cm SSD with a 30 cm × 30 cm field and a nominal dose rate of 800 MU/min to determine the depth of maximum ionization at which the output factors were measured. The ion chamber was scanned at a speed of 0.5 cm/s and the W2 scintillator at a speed of 0.25 cm/s to a depth of 25 cm in the 3D Scanner water tank. The sampling speed was chosen based on the size of the detector to balance the time needed for each measurement with the signal-to-noise ratio. The data were analyzed with SNC Dosimetry software version 3.3.1.23121 (Sun Nuclear Corporation). The PDDs were smoothed with iterative denoising and normalized to the depth of maximum dose (d_max_). The PDDs measured by the ion chamber were shifted upstream by 0.6 r_cav_ according to the guidelines of AAPM TG-51 protocol and its addendum [22,26].

In-plane and cross-plane profiles were measured for 1 cm × 1 cm, 3 cm × 3 cm, 5 cm × 5 cm, and 10 cm × 10 cm fields at 100 cm SSD at d_max_ with the CC01 ion chamber and the W2 scintillator. Profile data were processed in SNC Dosimetry software. The raw data from both detectors were smoothed with the adaptive data density technique, centered, normalized to the central axis, and interpolated to 0.1 cm resolution.

We measured the output constancy of each detector as a function of pulse repetition frequency (PRF) and DPP. In a pulsed beam delivery, the electrons are accelerated in bunches, resulting in short bursts of beam spread out over a short time interval. The dose rate that is reported on the linear accelerator control panel is the average nominal dose rate in monitor units per minute (MU/min), where 1 MU is 1 cGy under calibration conditions (d_max_, 100 cm SSD, 10 cm × 10 cm field size). However, the instantaneous dose rate during each pulse is much higher than the nominal dose rate. To lower the nominal dose rate, fewer pulses were delivered over a given time, but the DPP was constant at 0.13 MU/pulse.

The PRF was varied by changing the nominal dose rate in the beam delivery settings. DPP was varied by moving the detector and phantom closer to the source, decreasing the SSD from 100 cm to 80 cm. We used output factors from the commissioning of the machine to account for the differences from reference conditions in field size, scatter factors, and the inverse square factor [27]. DPP at d_max_ for a 30 cm × 30 cm field is 0.14, 0.16, and 0.20 cGy for SSDs of 100 cm, 90 cm, and 80 cm, respectively. All measurements were performed with the collimator set to a 30 cm × 30 cm field, and the center of each detector was placed at the depth of maximum ionization according to the measured PDD of that chamber. Three readings from the delivery of 200 MU were averaged at nominal dose rates of 400, 800, 1200, 1600, 2000, and 2400 MU/min at 100 cm, 90 cm, and 80 cm SSD. For analysis, the data for each detector were normalized to the reading at 100 cm SSD and 800 MU/min.

## 3. Results

### 3.1. P_ion_ and P_pol_

The P_pol_ values for the CC08 were determined to be 0.998, 0.997, and 1.001 at SSDs of 80 cm, 90 cm, and 100 cm, respectively. For the CC01 the P_pol_ values were 0.977, 1.002, and 0.993 at SSDs of 80 cm, 90 cm, and 100 cm, respectively. P_pol_ is not expected to vary with DPP, which is consistent with our observations [2]. For the CC08 ion chamber, P_ion_ was measured to be 1.032, 1.040, and 1.048 at SSDs of 100 cm, 90 cm, and 80 cm, respectively. For the CC01 ion chamber, P_ion_ was measured to be 1.000, 1.005, and 1.008 at SSDs of 100 cm, 90 cm, and 80 cm, respectively. An increase in P_ion_ with SSD is expected, as ion recombination increases with increasing DPP. At 80 cm SSD, the P_ion_ for the CC08 approaches the limit of acceptability for reference dose measurements (1.05) recommended by the AAPM TG-51 report [22].

### 3.2. PDD Measurements

**Figure 2:**
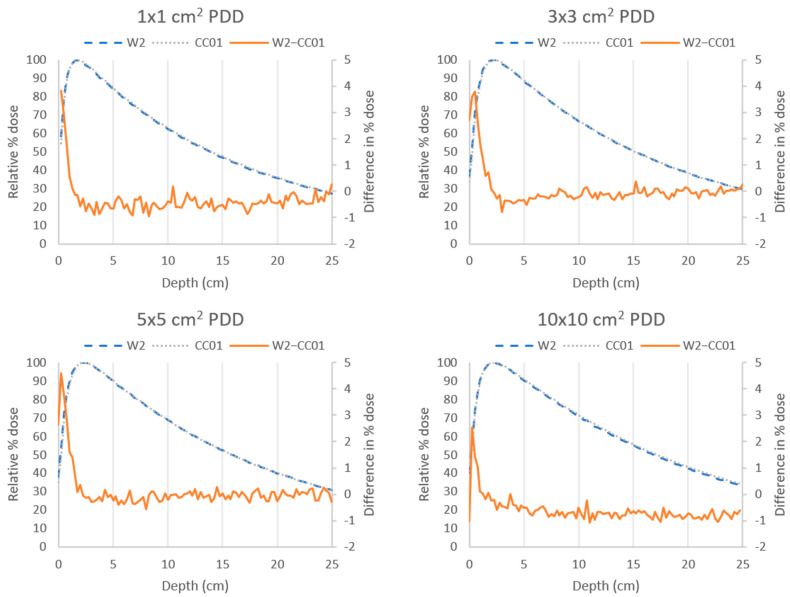
Percent depth dose (PDD) curves measured with the Exradin W2 scintillator (blue) and the CC01 ion chamber (gray) using a 10 MV flattening-filter-free (FFF) beam at 100 cm source-to-surface distance for various field sizes. The difference between the two measurements (W2—CC01, orange) was less than 5% at all points and less than 1% at depths greater than d_max_.

The PDD curves for field sizes of 1 cm × 1 cm, 3 cm × 3 cm, 5 cm × 5 cm, and 10 cm × 10 cm were measured with the W2 scintillator (blue curve) and CC01 ion chamber (gray curve) and are shown in Figure 2. The curves for both detectors agreed within 5% at depths shallower than d_max_ (build-up region) for all field sizes and within 1% at depths greater than d_max_. Similar agreement was seen between the CC08 ion chamber and the W2 scintillator (results not shown).

**Figure 3:**
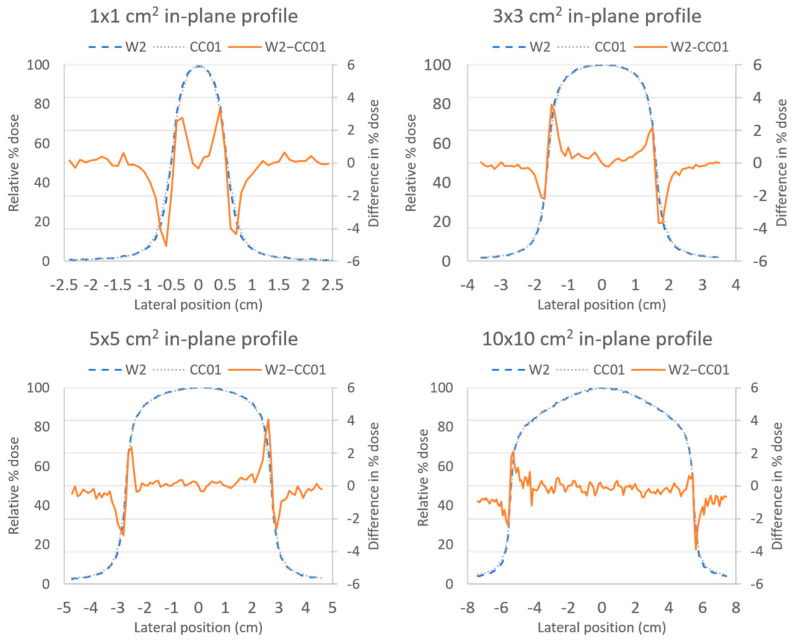
In-plane profiles measured with the Exradin W2 scintillator (blue) and the CC01 ion chamber (gray) using a 10 MV FFF beam at 100 cm source-to-surface distance for various field sizes. The difference between the two measurements (W2–CC01, orange) was less than 5% at all points and less than 1% at depths outside of the penumbra region.

The in-plane profile measurements shown in Figure 3 illustrated a similar agreement between the CC01 ion chamber and the W2 scintillator to that found for the PDD measurements. Agreement was within 5% inside the penumbra region, and within 1% outside of the penumbra region. Cross-plane profile measurements showed similar agreement (not shown here).

**Figure 4:**
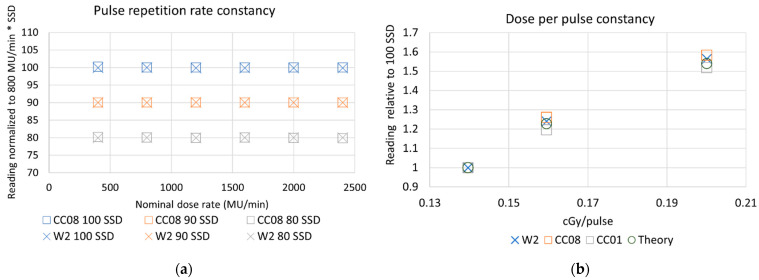
(**a**) Output of the CC08 ion chamber (squares) and W2 scintillator (X) is constant as a function of nominal dose rate at 100 cm SSD (blue, top row), 90 cm SSD (orange, middle row), and 80 cm SSD (gray, bottom row). Each detector’s output was normalized to the reading from the respective detector and SSD at 800 MU/min. (**b**) Detector signal as a function of DPP for the W2 scintillator, and CC08 and CC01 ion chambers, relative to the signal at 100 SSD (0.14 cGy/MU). The theoretical relationship that was expected is shown with circles.

The dose rate constancy of the W2 scintillator was investigated as a function of PRF and DPP. The results are summarized in Figure 4. The reading at each dose rate normalized to the reading at 800 MU/min is presented on the *y*-axis, multiplied by SSD for visual separation, and nominal dose rate is shown on the *x*-axis.

Theoretical values for detector response were calculated relative to the response at 100 cm SSD, accounting for changes in the field size, scatter factors, and inverse square factors. As shown in Figure 4a,b, all detectors had consistent output as a function of PRF or nominal dose rate (CC01 data not shown). This is as expected, since recombination effects are dependent on DPP, not PRF [2].

The detectors differed as a function of DPP, which was modulated by adjusting the SSD. The CC08 ion chamber response, shown in orange in Figure 4b, was 2.74% and 2.86% higher than expected from the theoretical calculations at 90 cm SSD (0.16 cGy/pulse) and 80 cm SSD (0.20 cGy/pulse), respectively. The W2 scintillator also showed a higher signal compared to the theoretical calculations, producing a signal 1.3% and 1.7% higher at 90 cm SSD (0.16 cGy/pulse) and 80 cm SSD (0.20 cGy/pulse), respectively. The smallest ion chamber, the CC01, showed a lower signal of −2.53% and −1.34% at 90 cm SSD (0.16 cGy/pulse) and 80 cm SSD (0.20 cGy/pulse), respectively.

## 4. Discussion

In this work, we investigated the dose rate linearity of the Exradin W2, a novel scintillation detector system, in a 10 MV FFF beam. Our PDD measurements showed good agreement between the CC08 ion chamber and the W2 scintillator at depths beyond the buildup region. The largest differences occurred in the buildup region and are likely due to small errors in detector positioning, which will have a large effect on measurements in the buildup region where the dose changes rapidly with depth [21]. A custom adaptor for the W2 to fit in holders designed for common ion chambers is usually included with the W2 system but was not available for this study. The correct use of this adapter should improve the precision of the detector positioning.

Other authors have found that the decrease in P_ion_ at greater depths should be accounted for in relative dosimetry measurements [3,28]. This correction was not applied in this work and the value of P_ion_ at d_max_ was used for the entire depth dose curve. This may have caused a small over-response from the ion chamber that increased with depth and may explain the difference between the curves at greater depths.

For the profile measurements, differences were largest in the penumbra region. These differences are due to volume averaging effects seen in the ion chamber measurements that were not seen in the W2 scintillator because of its small size. Galavis et al. [20] validated the measurement of small field profiles with the W2 scintillator and film for a 6 MV flattened beam. Our results for the 1 cm × 1 cm field profile at d_max_ agree with their findings. Such agreement is expected because the flattening filter should have a negligible effect on the profiles of very small fields [2].

Additionally, we found that P_ion_ increased linearly with DPP. It reached a maximum of 1.048 at 0.20 cGy/pulse with the CC08 ion chamber. This is close to the limit of 1.05 set out by the TG51 protocol to define a reference class ion chamber [22,26]. P_ion_ was much smaller for the CC01 ion chamber due to the smaller volume available to the electrons to recombine before reaching the electrode. Others have also shown a linear relationship between DPP and P_ion_ for other chambers [2,3]. The values we found are higher than those reported by others, but this could be attributed to the different construction of the detectors [2]. P_pol_ was not expected to vary with DPP, which is consistent with our observations [2].

The central finding of this work demonstrates that the W2 scintillator performs with similar accuracy to two small-volume ion chambers in high DPP conditions. Since the W2 scintillator does not rely on dose-rate-dependent correction factors like ion chambers do and can provide instantaneous dosimetric feedback unlike film, the W2 scintillator will be a valuable tool for dosimetry of high-dose-rate beams. The small volume of the W2 scintillator also makes it ideal for scanning and small field dosimetry.

## 5. Conclusions

The Exradin W2 scintillator is a newly available detector designed specifically for small field scanning. We evaluated the dose rate constancy of the W2 scintillator and compared measurements of profiles and PDDs to two ion chambers in a 10 MV FFF beam. We found excellent agreement between the chambers and the W2 scintillator for scanning and relative dosimetry measurements.

## Figures and Tables

**Table 1:** Physical properties of the detectors used and compared.

Detector	Dimensions: Length, Inner Diameter (mm)	Active Volume (cm^3^)	Detector Material
CC01 [23]	3.6, 2.0	0.01	0.35 mm diameter steel electrode in air
CC08 [24]	4.0, 6.0	0.08	1 mm diameter C552 electrode in air
Exradin W2 [25]	1.0, 1.0	0.0008	Solid polystyrene with polyimide stem

## Data Availability

Not applicable.
